# Genome analysis of American minks reveals link of mutations in *Ras-related protein-38* gene to Moyle brown coat phenotype

**DOI:** 10.1038/s41598-020-72239-5

**Published:** 2020-09-28

**Authors:** Andrey D. Manakhov, Maria Yu. Mintseva, Igor A. Andreev, Lev I. Uralsky, Tatiana V. Andreeva, Oleg V. Trapezov, Evgeny I. Rogaev

**Affiliations:** 1 Department of Genomics and Human Genetics, Vavilov Institute of General Genetics, Russian Academy of Sciences, Moscow, Russia 119333; 2 Center for Genetics and Genetic Technologies, Faculty of Biology, Lomonosov Moscow State University, Moscow, Russia 119192; 3 Sirius University of Science and Technology, Sochi, Russia 354340; 4 Department of Animals and Human Genetics, Institute of Cytology and Genetics, Siberian Branch of the Russian Academy of Sciences, Novosibirsk, Russia 630090; 5 Novosibirsk State University, Novosibirsk, Russia 630090; 6 Department of Psychiatry, University of Massachusetts Medical School, Worcester, MA 01604 USA

**Keywords:** Genome, Genomics, Genotype, Mutation, Sequencing

## Abstract

Over 35 fur colours have been described in American mink (*Neovison vison*), only six of which have been previously linked to specific genes. Moyle fur colour belongs to a wide group of brownish colours that are highly similar to each other, which complicates selection and breeding procedures. We performed whole genome sequencing for two American minks with Moyle (*m/m*) and Violet (*a/a m/m /p/p*) phenotypes. We identified two frame-shift mutations in the gene encoding Ras-related protein-38 (*RAB38*), which regulates the trafficking of tyrosinase-containing vesicles to maturing melanosomes. The results highlight the role of *RAB38* in the biogenesis of melanosomes and melanin and the genetic mechanism contributing to hair colour variety and intensity. These data are also useful for tracking economically valuable fur traits in mink breeding programmes.

## Introduction

Coat colour polymorphism is a well-known phenomenon in mammals^1^. Humans have been collecting animals with unusual coat colours and breeding them to create new colour variations for a long time. This selection results in animals with fur colours that are not present in natural populations. The American mink is an amazing example of this. More than one century of artificial selection of the American mink resulted in a wide spectrum of colour variation^2,3^. Only 6 of the greater than 30 mutations affecting fur colour have been linked to specific DNA mutations^4–8^.

Among the diverse fur colours of the American mink, a number of phenotypes exhibit a range of brownish colours. At least 12 phenotypes have a brown appearance in a wide range from light brown [American palomino (*k/k*)] to chocolate [Moyle (*m/m*)] and dark brown [pastel (*b/b*), and standard dark brown (+*/*+)]. All brown phenotypes are inherited as recessive Mendelian traits and seem to be the result of mutations in different genomic loci. These phenotypes are highly similar to each other, which significantly complicates selection and breeding^7^.

**Figure 1:**
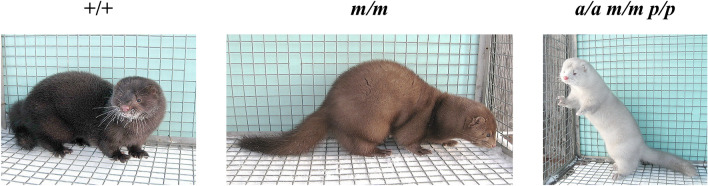
American minks of standard dark brown, Moyle (*m/m*) and Violet (*a/a m/m p/p*) phenotypes.

Moyle is a light brown mink coat colour (Fig. 1). This phenotype is inherited as a Mendelian autosomal recessive trait, and three alleles were postulated: Moyle (*m*), Cameo (*m*^*c*^), and wild type (+). The Cameo allele produces a darker shade of brown than the Moyle allele, and seems dominant to it. Both alleles are recessive to wild type, which is a standard dark brown colour. (+ > *m*^*c*^ > *m*)^3^. The Moyle mutation is widespread in the mink fur industry, and it is used in combination with other mutations to generate popular fur colours, such as Lavender (*a/a m/m*), Moyle platinum (*m/m p/p*) and Violet (*a/a m/m p/p*)^2^.

The present study investigated the genetic factors that determine the Moyle phenotype using whole-genome sequencing of minks with Moyle (*m/m*) and Violet (*a/a m/m /p/p*) fur colour (Fig. 1).

## Results

To the best of our knowledge, the present study is the first study to perform whole genome sequencing of American minks with Moyle (*m/m*) and Violet (*a/a m/m p/p*) fur colours. The resulting genome coverages were × 9 and × 40, respectively. We also used whole genome sequencing data of 3 standard dark brown (the mean genome coverage is × 8) and 3 Silverblue minks (the mean genome coverage is × 5) from our previous study^8^ (Supplementary Table 1).

### Mutations in the *RAB38* gene

We used GATK software^9^ to predict SNPs and InDels in sequenced mink genomes. We identified 13,827,261 variants across all genomes. To detect the genetic factor underlying the Moyle phenotype, we selected homozygous or compound heterozygous variants in Moyle (*m/m*) and Violet (*a/a m/m p/p*) minks that were not homozygous or compound heterozygous in standard dark brown (+*/*+) and Silverblue (*p/p*) animals. Among the selected variations, we found two homozygous mutations (one in the Moyle sample and one in the Violet sample) in Ras-related protein 38 gene (*RAB38*). Both mutations had a putative “HIGH” impact based on the VEP^10^ prediction.

**Figure 2:**
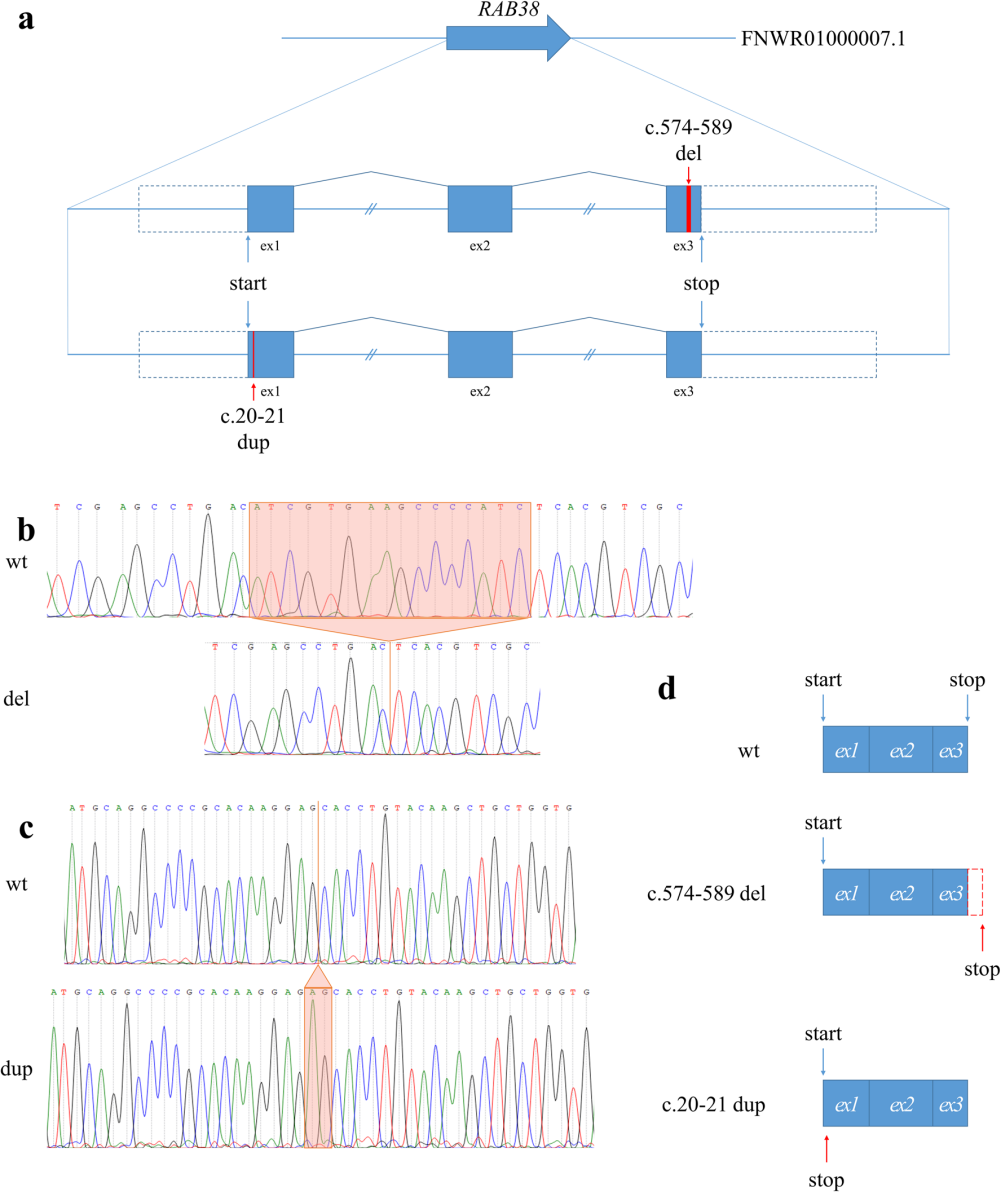
Effects of *RAB38*^*3del*^ and *RAB38*^*1dup*^ mutations on *RAB38* transcripts. (**a**) Structure of the *RAB38* gene. Red boxes indicate *RAB38*^*3del*^ and *RAB38*^*1dup*^ mutations. Equal intron sizes are shown for simplification. **b.** An electrophoregram of Sanger sequencing for *RAB38* gDNA exon 3. The orange frame is a 16-bp deletion in Moyle (*m/m*) minks with homozygous *RAB38*^*3del*^ mutation. (**c**) An electrophoregram of Sanger sequencing for *RAB38* gDNA exon 1. The orange frame is the 2-bp duplication in Violet (*a/a m/m p/p*) minks with homozygous *RAB38*^*1dup*^ mutation. (**d**) Effects of *RAB38*^*3del*^ and *RAB38*^*1dup*^ mutations on *RAB38* transcripts.

We identified a homozygous 16-bp deletion [FNWR01000007.1:16075438–16075453del (RAB38:c.574-589del)], hereinafter referred to as *RAB38*^*3del*^, in the Moyle sample, at the third exon of *RAB38* gene. We found a homozygous 2-bp duplication [FNWR01000007.1:16132224_16132225dupCT (RAB38:c.20-21dup)], hereinafter referred to as *RAB38*^*1dup*^, in the Violet sample, at the first exon of the *RAB38* gene. Both mutations potentially resulted in the loss of function of RAB38 protein (Fig. 2).

We used Sanger sequencing and found that all 3 Moyle (*m/m*) minks were homozygous for *RAB38*^*1dup*^ mutations or were heterozygous for both the *RAB38*^*3del*^ and *RAB38*^*1dup*^ mutations. The single Lavender mink (*a/a m/m*) was also heterozygous for both mutations. Among four Violet minks (*a/a m/m p/p*) two were homozygous for *RAB38*^*3del*^ mutation, one was homozygous for *RAB38*^*1dup*^ and one was heterozygous for both (Table 1).

**Table 1:** Results of *RAB38*^*3del*^ and *RAB38*^*1dup*^ genotyping in American mink.

Symbol	Scandinavian phenotype name	Genotype *RAB38*^*1dup*^	Genotype *RAB38*^*3del*^	N samples
*m/m*	Moyle	+/+	del/del	1
		+/dup	+/del	2
*a/a m/m*	Lavender	+/dup	+/del	1
*a/a m/m p/p*	Violet	dup/dup	+/+	1
		+/dup	+/del	1
		+/+	del/del	2
+*/*+	Standard dark brown	+/+	+/+	17
*p/p*	Silverblue	+/+	+/+	2
*S*^*H*^*/*+ *p/p*	Shadow silverblue	+/+	+/+	1
*b/b*	Royal pastel	+/+	+/+	5
		+/+	+/del	1
*S/*+ *a/a p/p*	Cross sapphire	+/+	+/+	1

Twenty-six of the 27 standard brown or wild-type minks, and minks with other colour coats not postulated to have a Moyle allele, were homozygous wild type at the both tested mutations (Table 1), and a single mink was heterozygous for the *RAB38*^*1dup*^ mutation.

Allele-specific reverse transcription polymerase chain reaction (RT-PCR) was performed to confirm the chromosome location of *RAB38*^*3del*^ and *RAB38*^*1dup*^ mutations in double heterozygotes animals. Sequencing of the allele-specific cDNA amplicons encompassing exons 1–3 revealed that the *RAB38*^*3del*^ and *RAB38*^*1dup*^ mutations in double heterozygote animals were located on different chromosomes (Supplementary Fig. 2).

Taken together, our data suggest that mutations *RAB38*^*3del*^ and *RAB38*^*1dup*^ are associated with the Moyle fur colour phenotype.

## Discussion

The *RAB38* gene encodes the member of the Rab small G protein family, which is involved in intracellular vesicle trafficking and melanosome biogenesis^11^. The *RAB38* gene is highly expressed in melanocytes, and RAB38 protein co-localizes with end-stage melanosomes^12,13^. RAB38 participates in the transport of newly synthesized tyrosinase and Tyrp1, which are key enzymes in melanin production from the trans-Golgi network endosomes to maturing melanosomes^14,15^. Mutations in *RAB38* gene were previously described in the dilution of coat colour in *chocolate* (*cht*) mice^12^ and *Ruby* rats^13^.

**Figure 3:**
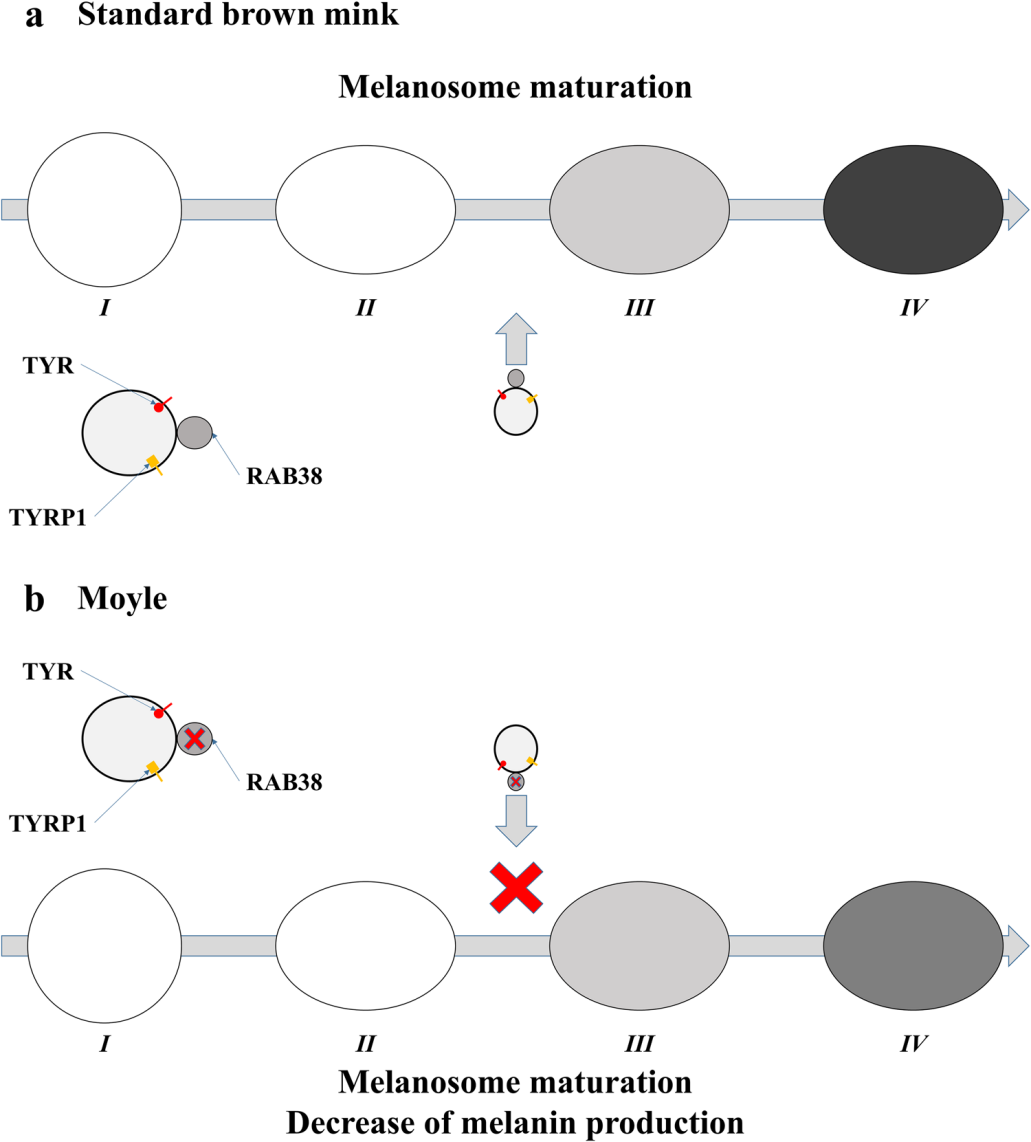
Schematic of *RAB38* function during melanosome maturation trafficking in standard dark brown (**a**) and Moyle (**b**) minks. RAB38 interacts with the membrane of vesicles containing newly synthesized tyrosinase (red circle) and Tyrp1 (orange square) and activates its transport to maturing melanosomes. I, II, III, and IV is states of melanosome maturation.

The *RAB38*^*3del*^ mutation found in mink may result in a frame shift at the 192 protein position and lead to the loss of a stop-codon at the 212 position. A novel potential stop codon occurs only at the 277 protein position, which results in a 30% enlargement of protein size and a C-terminal end that is completely different from wild-type (Fig. 2). The mutant protein loses the C-terminal-interacting motif (amino acids 193–195), which is conserved in the RAB protein family, and seems to be involved in the interaction with Rab escort protein^16^. The mutant protein also lacks the C-terminal cysteine (position 208) within the geranylgeranylation motifs. This cysteine is the substrate for the covalent attachment of geranylgeranyl moieties, and it is highly important for RAB protein function, and present in all members of the RAB protein family^16^. Geranylgeranylated RAB proteins are inserted in a regulated manner into specific membranes where it interacts with GDI displacement factor, guanine nucleotide exchange factor and Rab effectors to control vesicular trafficking events^16,17^. We hypothesized that the mutant RAB38 protein had lower efficiency and/or specificity to interact with vesicles containing tyrosinase and Tyrp1, which would resulted in a decrease of melanin production and lead to the fur colour dilution from dark to light brown (Fig. 3).

The *RAB38*^*1dup*^ mutation may result in a frame shift at the 8 protein position and lead to a premature stop-codon at the 15 position (Fig. 2).

We suggest that both of the identified mutations in *RAB38* are causative for the Moyle (*m*/*m*) phenotype in the Novosibirsk mink population. Since at least two alleles Moyle (*m*) and Cameo (*m*^*c*^) were described as autosomal recessive to standard dark brown (+)^3^ it can be hypothesized that different allelic mutations in *RAB38* gene may underlie the phenotype variabilities linked to the same locus. This suggestion has yet to be tested by direct *RAB38* gene genotyping of Cameo animals.

Our study adds the *RAB38* gene to the list of genes with identified mutations which were associated with different mink fur colour phenotypes^4–8^ and provides valuable data that contribute to improving global mink fur production via selective breeding programs.

## Methods

All methods were carried out in accordance with relevant guidelines and regulations for laboratory work. The local Ethics Committee of the Institute of Cytology and Genetics, Siberian Branch of the Russian Academy of Sciences, approved the study protocols.

Moyle (*m/m* 3 individuals), lavender (a/a m/m 1 individuals), violet (*a/a m/m p/p* 4 individuals), Silverblue (*p/p*, 2 individuals), royal pastel (*b/b* 2 individuals), shadow silverblue (*S*^*H*^*/*+ *p/p* 1 individual) and standard dark brown (+/+ 17 individuals) farm-bred American minks were maintained in the Experimental Fur Farm of the Institute of Cytology and Genetics (Novosibirsk mink population). The collected tissues (tail snips) were rapidly dissected, frozen in liquid nitrogen, and stored at − 70 °C until DNA and RNA extraction.

Sample collections from farm-bred American minks of royal pastel (*b/b* 4 individuals) and Cross sapphire (*S/*+ *a/a p/p* 1 individual) coat colour from the «Mermeriny» fur farm, Tver region, Russia (Tver mink population) were used in this study. The Tver mink population is unrelated to the Novosibirsk population.

Genomic DNA from mink tissues was extracted using QIAGEN Mini Spin Columns, following the manufacturer’s protocol (QIAGEN, Germany). Library preparations (1 Moyle (*m/m*) and 1 violet (*a/a m/m p/p*) were performed using the TruSeq PCR Free Kit (Illumina, USA), following the manufacturer’s protocol. Library validation was performed using an Agilent 2,100 Bioanalyzer with DNA High Sensitivity chip (Agilent, USA) and quantified via qPCR using a KAPA Library Quantification Illumina Kit protocol (KAPA Biosystems, USA). Paired-end libraries were sequenced in 2 × 101 cycles using the Illumina TruSeq SBS v3 kit (Illumina) on a HiSeq 2,000/2,500 sequencer (Illumina) at the Vavilov Institute of General Genetics RAS (Moscow, Russia) and 2 × 151 cycles using the Illumina NovaSeq S4 kit (Illumina) on a NovaSeq 6,000 sequencer at the Genetico Company (Moscow, Russia).

We also used whole genome sequencing data of 3 standard dark brown Silverblue minks from our previous study^8^.

The resulting reads were mapped to the American mink (*Neovison vison*) genome (NNQGG.v01) using a BWA-MEM algorithm (bwa v.0.7.13-r112)^18^. Duplicate reads were detected using the MarkDuplicates algorithm from picard-tools v.1.133 (broadinstitute.github.io/picard) and excluded from further analyses.

Genetic variants in the sequenced mink genomes were predicted using HaplotypeCaller (with default arguments) from the Genome Analysis Toolkit (GATK) package version 4.0^9^.

To detect the genetic factor underlying the Moyle phenotype, we selected homozygous or compound heterozygous variants with a depth of coverage greater than 2 in *m/m* and *a/a m/m p/p* minks that were not homozygous or compound heterozygous in the standard dark brown wild-type and Silverblue animals.

Annotation and effect prediction of selected variants were performed in VEP^10^ using the American mink genome annotation (Ensembl v97).

We performed Sanger sequencing to validate mutations. Primers for PCR amplification were designed in Primer3 software (Supplementary Table 2), and PCR was performed using the GenPack PCR Core (Isogen, Russia). Resultant amplicons were cleaned using a Cleanup Standard Kit (Evrogen, Russia) and processed using the BigDye Terminator v3.1 Cycle Sequencing Kit (Applied Biosystems, USA), following the manufacturers’ protocols. Probes were purified using a DyeEx 2.0 Spin Kit (QIAGEN) and sequenced in a 3730xl DNA Analyzer (Applied Biosystems).

To identify the chromosome location of *RAB38*^*3del*^ and *RAB38*^*1dup*^ mutations in double heterozygotes animals, we used allele-specific RT-PCR (Supplementary Fig. 1). Total RNA was extracted from tissues using RNeasy Mini Spin Columns, following the manufacturer’s protocol (QIAGEN). Extracted RNA was treated with RNase-Free DNase I (Thermo Scientific, USA) and assayed for quantity and quality in a NanoDrop One-C (Thermo Scientific). All RNA samples were kept at − 80 °C. First-strand cDNA synthesis was performed using the High-Capacity cDNA Reverse Transcription Kit (Applied Biosystems). The first amplification was performed using universal cDNA RAB38 ex 1–3 F and cDNA RAB38 ex 1–3 R primers. The resulting PCR products were used for the second amplification with (1) universal cDNA RAB38 ex 1–3 F and cDNA RAB38 ex 1–3 R primers, (2) wt-specific primers cDNA RAB38 ex 1–3 F and cDNA RAB38 ex 1–3 wt R, and (3) del-specific primers cDNA RAB38 ex 1–3 F and cDNA RAB38 ex 1–3 del R. Final PCR products were cleaned up using a Cleanup Standard Kit (Evrogen) and processed using the BigDye Terminator v3.1 Cycle Sequencing Kit (Applied Biosystems), following the manufacturers’ protocols. Probes were purified using a DyeEx 2.0 Spin Kit (QIAGEN) and sequenced in a 3730xl DNA Analyzer (Applied Biosystems).

## Supplementary information

Supplementary Information.

## Data Availability

The datasets generated during the current study were deposited into NCBI SRA database and can be accessed with the BioProject accession number PRJNA660737 (https://www.ncbi.nlm.nih.gov/sra/PRJNA660737).

## References

[1] Hofreiter M, Schöneberg T (2010). The genetic and evolutionary basis of colour variation in vertebrates. Cell. Mol. Life Sci..

[2] Tpaпeзoв OB, Tpaпeзoвa ЛИ (2009). Bocпpoизвoдящaяcя кoллeкция oкpacoчныx гeнoтипoв aмepикaнcкoй нopки (*Mustela vison* Schreber, 1777) нa экcпepимeнтaльнoй звepoфepмe Инcтитyтa цитoлoгии и гeнeтики CO PAH. (A reproducing collection of American mink (Mustela vison Schreber, 1777) color genotypes at the experimental fur farm of the Institute of cytology and genetics, Novosibirsk). Becтник BOГиC.

[3] Robinson R, King RC (1975). The American Mink, Mustela vison. Handbook of Genetics.

[4] Anistoroaei R, Fredholm M, Christensen K, Leeb T (2008). Albinism in the American mink (Neovison vison) is associated with a tyrosinase nonsense mutation. Anim. Genet..

[5] Benkel BF, Rouvinen-Watt K, Farid H, Anistoroaei R (2009). Molecular characterization of the Himalayan mink. Mamm. Genome.

[6] Anistoroaei R, Krogh AK, Christensen K (2013). A frameshift mutation in the LYST gene is responsible for the Aleutian color and the associated Chediak-Higashi syndrome in American mink. Anim. Genet..

[7] Cirera S (2016). A large insertion in intron 2 of the TYRP1 gene associated with American Palomino phenotype in American mink. Mamm. Genome.

[8] Manakhov AD, Andreeva TV, Trapezov OV, Kolchanov NA, Rogaev EI (2019). Genome analysis identifies the mutant genes for common industrial Silverblue and Hedlund white coat colours in American mink. Sci. Rep..

[9] McKenna A (2010). The genome analysis toolkit: a MapReduce framework for analyzing next-generation DNA sequencing data. Genome Res..

[10] McLaren W (2016). The ensembl variant effect predictor. Genome Biol..

[11] Osanai K (2005). Expression and characterization of Rab38, a new member of the Rab small G protein family. Biol. Chem..

[12] Loftus SK (2002). Mutation of melanosome protein RAB38 in chocolate mice. Proc. Natl. Acad. Sci. USA.

[13] Oiso N, Riddle SR, Serikawa T, Kuramoto T, Spritz RA (2004). The rat Ruby (R) locus is Rab38: identical mutations in fawn-hooded and tester-moriyama rats derived from an ancestral long evans rat sub-strain. Mamm. Genome.

[14] Bultema JJ, Ambrosio AL, Burek CL, Di Pietro SM (2012). BLOC-2, AP-3, and AP-1 proteins function in concert with Rab38 and Rab32 proteins to mediate protein trafficking to lysosome-related organelles. J. Biol. Chem..

[15] Coppola U, Annona G, D’Aniello S, Ristoratore F (2016). Rab32 and Rab38 genes in chordate pigmentation: an evolutionary perspective. BMC Evol. Biol..

[16] Pylypenko O, Hammich H, Yu I-M, Houdusse A (2018). Rab GTPases and their interacting protein partners: structural insights into Rab functional diversity. Small GTPases.

[17] Shahbaaz M (2018). Structural insights into Rab21 GTPase activation mechanism by molecular dynamics simulations. Mol. Simul..

[18] Li H, Durbin R (2009). Fast and accurate short read alignment with Burrows–Wheeler transform. Bioinformatics.

