# Supplementary material for: Genome analysis of American minks reveals link of mutations in Ras-related protein-38 gene to Moyle brown coat phenotype
Source: Sci Rep. 2020 Sep 28;10:15876. doi: 10.1038/s41598-020-72239-5 (PMC7522971; doi:10.1038/s41598-020-72239-5)
Supplement: Supplementary file 1 — Supplementary Information. [file 41598_2020_72239_MOESM1_ESM.pdf]

Genome analysis of American minks reveals link of mutations in *Ras-related protein-38* gene to Moyle brown coat phenotype

Andrey D. Manakhov [1, 2, 3] (orcid: 0000-0002-5163-8747)

Maria Yu. Mintseva [1, 2] (orcid: 0000-0003-4613-471X)

Igor A. Andreev [2]

Lev I. Uralsky [1] (orcid: 0000-0002-5565-7961)

Tatiana V. Andreeva [1, 2]

Oleg V. Trapezov [4, 5]

Evgeny I. Rogaev [1, 2, 3, 6] *

1) Department of Genomics and Human Genetics, Vavilov Institute of General Genetics, Russian Academy of Sciences, Moscow, 119333, Russia
2) Center for Genetics and Genetic Technologies, Faculty of Biology, Lomonosov Moscow State University, Moscow, 119192, Russia
3) Sirius University of Science and Technology, Sochi, 354340, Russia
4) Department of Animals and Human Genetics, Institute of Cytology and Genetics, Siberian Branch of the Russian Academy of Sciences, Novosibirsk, 630090, Russia
5) Novosibirsk State University, Novosibirsk, 630090, Russia
6) Department of Psychiatry, University of Massachusetts Medical School, Worcester, MA 01604, USA

* Corresponding author (rogaev@vigg.ru; Vavilov Institute of General Genetics, Russian Academy of Sciences, Moscow, 119333, Russia; tel: 8 (499) 135-50-61)

Supplementary Table 1. Results of sequencing of American mink genomes. Statistics were calculated using samtools [1] and picard software. American mink genome (NNQGG.v01) was used as a reference.

Sample	***Colour***	***N of reads***	***Mapped %***	***Duplicates %***	***Coverage***
mink_7-331	*m/m*	242,268,164	97.72	0.72	9.06
mink_7-317	*a/a m/m p/p*	815,276,506	98.22	8.29	40.21
mink_3-261 [2]	+/+	261,394,248	97.65	3.99	9.04
mink_3-265 [2]	+/+	230,510,854	97.80	2.10	8.49
mink_3-247 [2]	+/+	252,307,168	98.54	2.61	7.07
mink_1-663 [2]	*p/p*	181,699,206	96.65	11.64	5.69
mink_0-329 [2]	*p/p*	167,858,388	98.09	2.96	5.41
mink_9-431 [2]	*p/p*	147,501,668	97.54	4.05	5.14

1. Li, H. *et al.* The Sequence Alignment/Map format and SAMtools. *Bioinformatics* **25**, 2078–2079 (2009).
2. Manakhov, A. D., Andreeva, T. V, Trapezov, O. V, Kolchanov, N. A. & Rogaev, E. I. Genome analysis identifies the mutant genes for common industrial Silverblue and Hedlund white coat colours in American mink. *Sci. Rep.* **9**, 4581 (2019).

Supplementary Table 2. Primer sequences used for cDNA and gDNA amplification.

Primer name	*Primer sequence*	*Expected amplicon size (bp)*	*Annealing t (°C)*
cDNA RAB38 ex 1 F<br>***cDNA RAB38 ex 3 R***	CGGGAAAGGAAGGAGGATTA<br>GCCTACTAGGATTTGGCACA	***836***	***60***
cDNA RAB38 ex 1 F<br>***cDNA RAB38 ex 3 wt R***	CGGGAAAGGAAGGAGGATTA<br>TGAGATGGGGCTTCACGA	***787***	***60***
cDNA RAB38 ex 1 F<br>***cDNA RAB38 ex 3 del R***	CGGGAAAGGAAGGAGGATTA<br>TCTTGGGCGACGTGAGTC	***783***	***60***
gDNA RAB38 ex 1 F<br>***gDNA RAB38 ex 1 R***	CTGCCTCTCTTCCAAGCTCT<br>GCACCAGGAAAAGTCTCGT	***838***	***58***
gDNA RAB38 ex 3 F<br>***gDNA RAB38 ex 3 R***	CAGATGCCTGGTGAAACATA<br>GCCTACTAGGATTTGGCACA	***132***	***58***

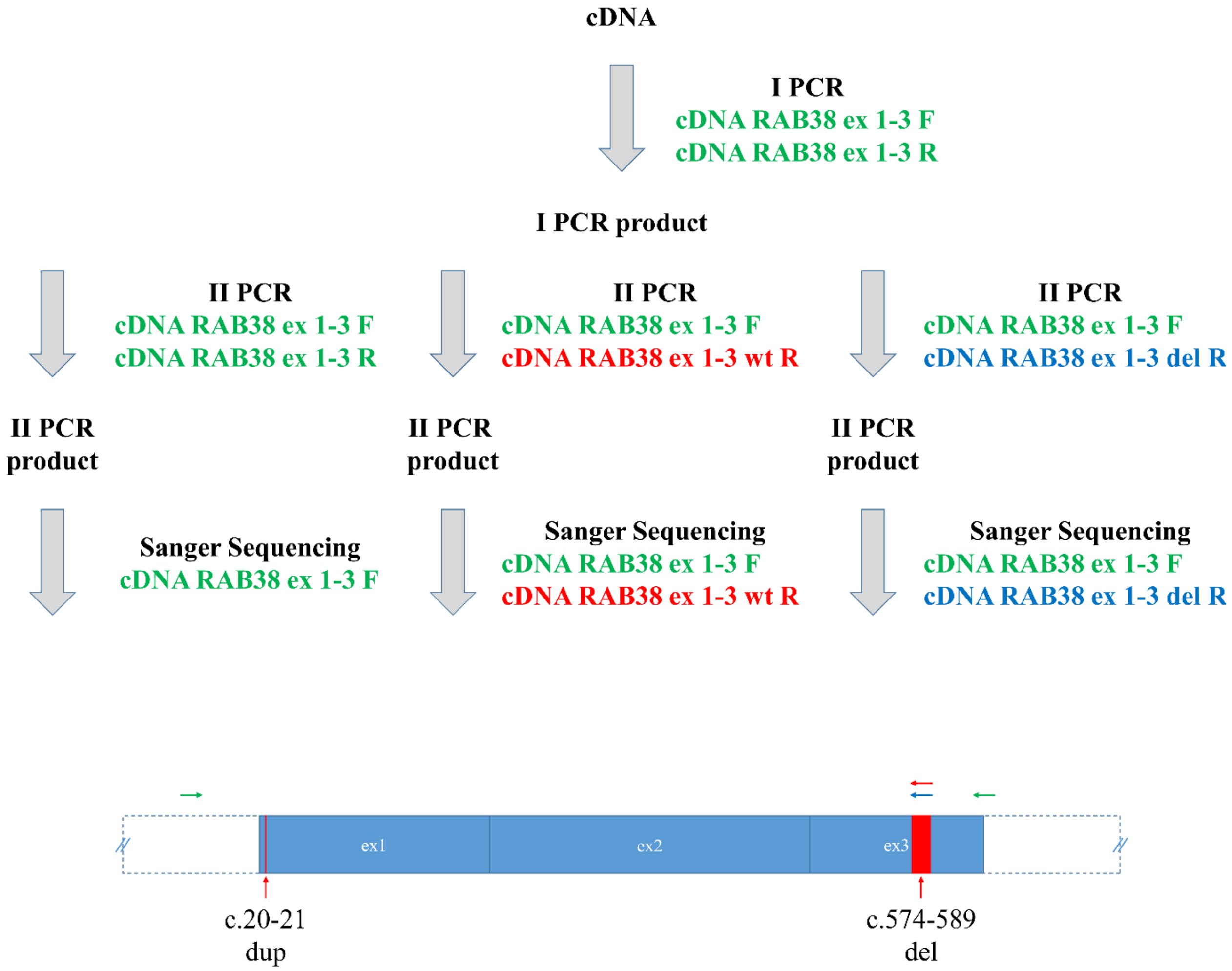

Supplementary Figure 1. *RAB38* allele-specific RT-PCR experimental design. Arrows indicate primer locations. Green indicate universal primers cDNA RAB38 ex 1-3 F and cDNA RAB38 ex 1-3 R primers, which were common for both alleles. Red is the allele-specific primer cDNA RAB38 ex 3 wt R. Blue is the allele-specific primer cDNA RAB38 ex 3 del R. Dotted boxes indicate 5'- and 3'-UTRs, and equal UTRs sizes are shown for simplification.

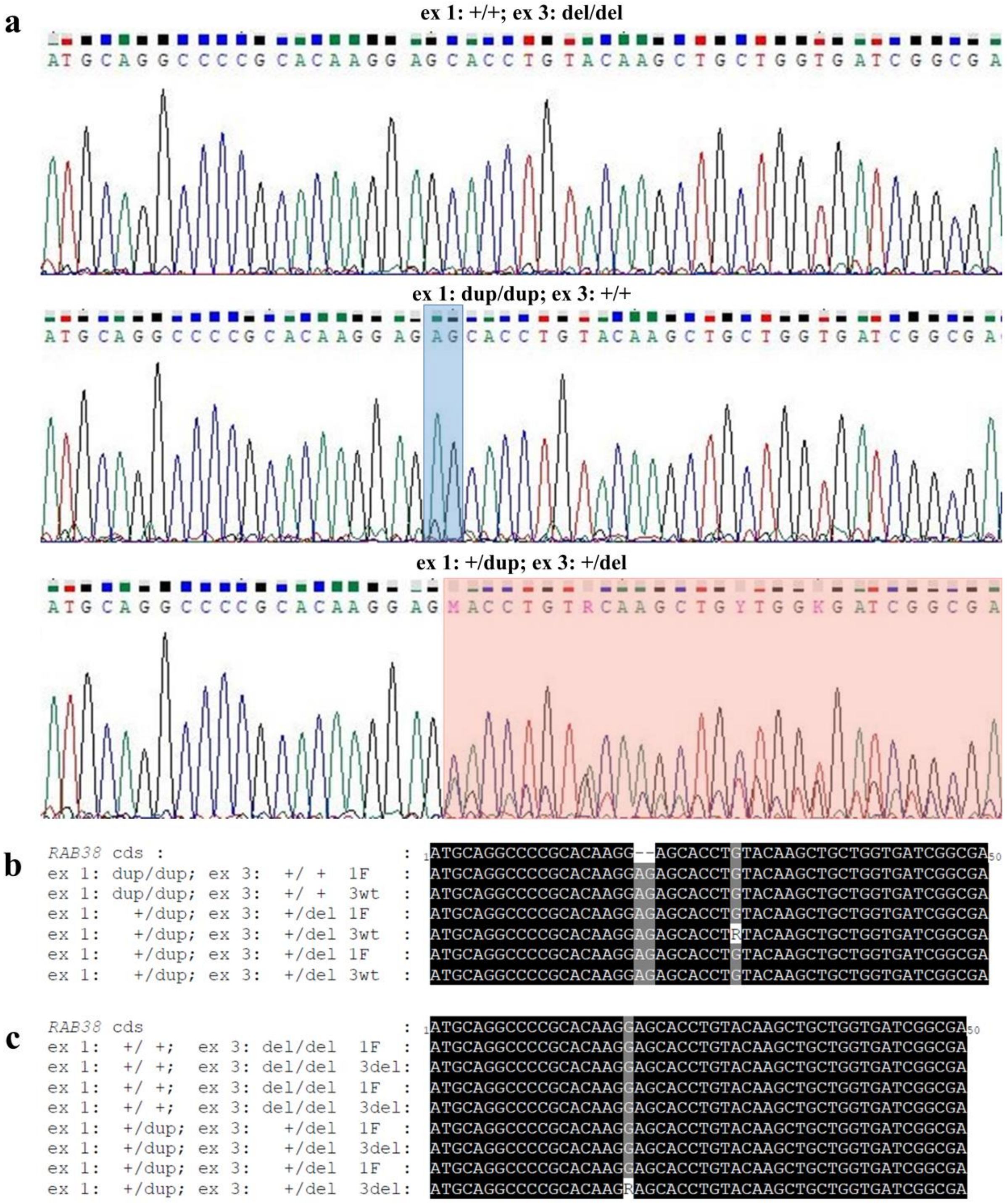

Supplementary Figure 2. Mutations of *RAB38*^{3del} and *RAB38*^{1dup} in double heterozygotes animals are located on different chromosomes. **a.** An electrophoregram of Sanger sequencing for cDNA II PCR amplicons (from primers cDNA RAB38 ex 1-3 F and cDNA RAB38 ex 1-3 R) of animals with different genotypes for *RAB38*^{3del} and *RAB38*^{1dup} mutations. The blue frame is a 2-bp duplication in ex 1 of *RAB38*. Two matrix are present in double heterozygote animals, as indicated in the pink frame. **b.** Alignment of Sanger sequencing for cDNA II PCR amplicons (from primers cDNA RAB38 ex 1-3 F and cDNA RAB38 ex 3 wt R) of animals with different genotypes for *RAB38*^{3del} and *RAB38*^{1dup} mutations. **c.** Alignment of Sanger sequencing for cDNA II PCR amplicons (from primers cDNA RAB38 ex 1-3 F and cDNA RAB38 ex 3 del R) of animals with different genotypes for *RAB38*^{3del} and *RAB38*^{1dup} mutations.
